# Sensory variation of landrace peas (*Pisum sativum* L.): Impacts of variety, location, and harvest year

**DOI:** 10.1002/fsn3.4287

**Published:** 2024-06-19

**Authors:** Magnus Westling, Matti Wiking Leino, Stefan Wennström, Åsa Öström

**Affiliations:** ^1^ The School of Hospitality, Culinary Arts and Meal Science Örebro University Grythyttan Sweden; ^2^ The Archaeological Research Laboratory, Department of Archaeology and Classical Studies Stockholm University Stockholm Sweden

**Keywords:** cultivated diversity, legumes, NordGen, plant based, sustainable gastronomy, terroir

## Abstract

The transition to more diversified protein sources presents legumes, such as peas, as excellent alternatives to animal protein. In light of this shift, understanding the sensory variation of pea genetic resources becomes crucial in broadening their appeal and promoting greater consumption. This study aimed to identify key factors influencing the sensory attributes of landrace peas, including variety (accession), location (geographical area of cultivation), and harvest year. Through a quantitative descriptive sensory analysis of six Swedish landrace pea accessions, cultivated over 1–2 years in three different Nordic countries, we analyzed the sensory attributes in detail and evaluated potential interactions between the pea accessions, their respective growing locations, and the varying harvest years. The results show that the sensory variation in the studied landrace pea accessions is primarily attributed to the chosen accession, despite the differences in location and harvest year. Notably, the results also reveal the potential impact of the location on the perceived *sourness* and *umami* taste of peas. These findings underscore the critical role of careful variety selection and breeding to enhance the sensory experience of peas, enabling the development of diverse pea‐based products that cater to consumer preferences.

## Graphical abstract

This study aimed to identify key factors influencing the sensory attributes of landrace peas, including variety (accession), location, and harvest year. The results show that the sensory variation in the studied landrace pea accessions is primarily attributed to the chosen accession, despite the differences in location and harvest year. These findings underscore the critical role of careful cultivar selection and breeding to enhance the sensory experience of peas, enabling the development of diverse pea‐based products that cater to consumer preferences.

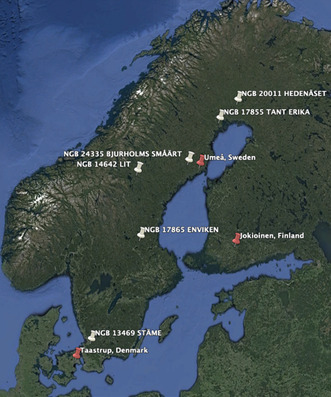

## 1 INTRODUCTION

Peas have a long history of cultivation in the Nordic countries, resulting in the evolution of a wide diversity of both field and garden pea varieties (Hagenblad et al., 2014; Leino et al., 2013; Solberg et al., 2015). These genetic resources are preserved at NordGen, an organization dedicated to safeguarding the common crop heritage of the Nordic countries and promoting the use of these genetic resources in cultivation and breeding efforts (NordGen, 2020). The “New Nordic Food Manifesto” emphasizes the importance of enhancing the sensory qualities of ingredients (Nordic Co‐operation, 2021). This emphasis not only offers chefs and food artisans a wide range of produce options but also fosters promising opportunities for exploring new culinary applications. Just as a painter relies on a wide array of colors to create their artwork, embracing a diversity of varieties provides culinary experts with a sensory palette to craft unique and distinctive dishes (McCauley, 2021).

Utilizing peas for culinary applications aligns with several sustainability aspects. Peas are rich in starch, protein, fiber, vitamins, minerals, and phytochemicals (Dahl et al., 2012). Although peas contain natural antinutrients, soaking and cooking them reduces these compounds (Wang et al., 2008), making the nutrients more available. Furthermore, the phytochemicals in peas, including polyphenolics, such as tannins (found in colored seed coat types as noted by Moïse et al., 2005), along with saponins and galactose oligosaccharides, contribute to various positive effects such as antioxidant, hypocholesterolemic, anticarcinogenic, and prebiotic effects (Dahl et al., 2012). Moreover, in terms of cultivation practices, peas naturally enrich the soil with nitrogen through symbiotic relationships with *Rhizobium* bacteria, minimizing the need for synthetic fertilizers and supporting a more resilient agricultural system. As peas can be cultivated in diverse climate zones, including subarctic climates, they present opportunities for local and regional food production. It is, therefore, evident that utilizing peas for culinary applications holds great potential for promoting sustainable food systems across diverse regions.

Although studies, such as the one conducted by Westling et al. (2019), have shed light on the sensory variations among different landraces and cultivars of peas, further research is needed to understand the potential impacts of location and year of harvest on these variations. Deepening our knowledge of the factors that influence the sensory quality of peas will allow us to fully unlock their gastronomic potential (Westling, 2022) while aligning with goals of sustainability and nourishment. Previous research has examined sensory variations in crops like apples, lettuce, and walnuts (Bunning et al., 2010; Lynch et al., 2016; Seppä et al., 2016). However, there is a lack of knowledge when it comes to landrace pea accessions and the impact of environmental factors on their sensory profiles. Consequently, this study aims to address this knowledge gap and contribute to a better understanding of sensory variations in landrace peas.

Landrace pea accessions are particularly interesting to study when considering gastronomic diversity in the Nordic countries. Alongside fava beans (*Vicia faba* L.), peas are the most common grain legumes cultivated in the region. In NordGen's project “Arctic peas – a potential protein source in the North,” a total of 50 different pea accessions were cultivated across Sweden, Finland, Norway, and Denmark over a 2‐year period (Carlson‐Nilsson et al., 2021; see Appendix: “Fifty pea cultivars” in Westling, 2022). This project aimed to explore the genetic resources of peas suitable for cultivation in the Nordic region, particularly in Arctic climates. One of the project's objectives was to highlight the existing variability in Nordic legumes. Building upon NordGen's project, our study serves as an important supplement, focusing on evaluating a selected subset of materials.

Specifically, our aim is to examine the individual and combined impacts of factors such as accession, location, and harvest year on the sensory variations in peas. Accession refers to the specific sample of different pea landraces, while the location encompasses factors such as temperature, precipitation, photoperiod, and soil. The harvest year reflects potential variations due to changing weather patterns and local growing conditions. By examining these factors, our objective is to gain a better understanding of how variety choice and environmental factors contribute to the overall sensory profiles of landrace peas. This will shed light on the potential influence of both genetic and environmental factors on the observed sensory variations.

## 2 MATERIALS AND METHODS

### 2.1 Material

#### 2.1.1 Plant material

Six specific pea accessions were chosen from an initial pool of 50 accessions (Carlson‐Nilsson et al., 2021) based on their superior overall yield. These selected accessions represent local landraces with diverse origins across various regions in Sweden and are preserved at NordGen (Table 1). Among the selected accessions, it is worth mentioning that “Lit” and “Stäme” are distinguished by their purple flowering, which contributes to pigmented seed test and a higher tannin content in comparison to white flowering peas.

**TABLE 1:** The six different pea accessions included in this study.

Accession	Name	Cultivar type	Color of flower	Seed type
NGB 13469	Stäme	Landrace	Purple	Field pea
NGB 14642	Lit	Landrace	Purple	Field pea
NGB 17855	Tant Erika	Landrace	White	Sugar pea
NGB 17865	Enviken	Landrace	White	Shelling pea
NGB 20011	Hedenäset	Landrace	White	Sugar pea
NGB 24335	Bjurholms småärt	Landrace	White	Field pea

The peas in this study are grouped based on flower color and three distinct seed types, as shown in Table 1. Field peas are primarily grown for mature seeds, which are dried and used in various culinary preparations like soups, stews, and purees. Shelling peas have large, edible seeds inside the pod. The pod itself is tough and inedible. Sugar peas have flat, edible pods that are bright green. They contain both immature peas and sweet, edible pods. Sugar peas are usually consumed whole, either raw or lightly cooked. However, for the purposes of this particular study, all peas were harvested at full maturity, when they had completely dried.

**FIGURE 1:**
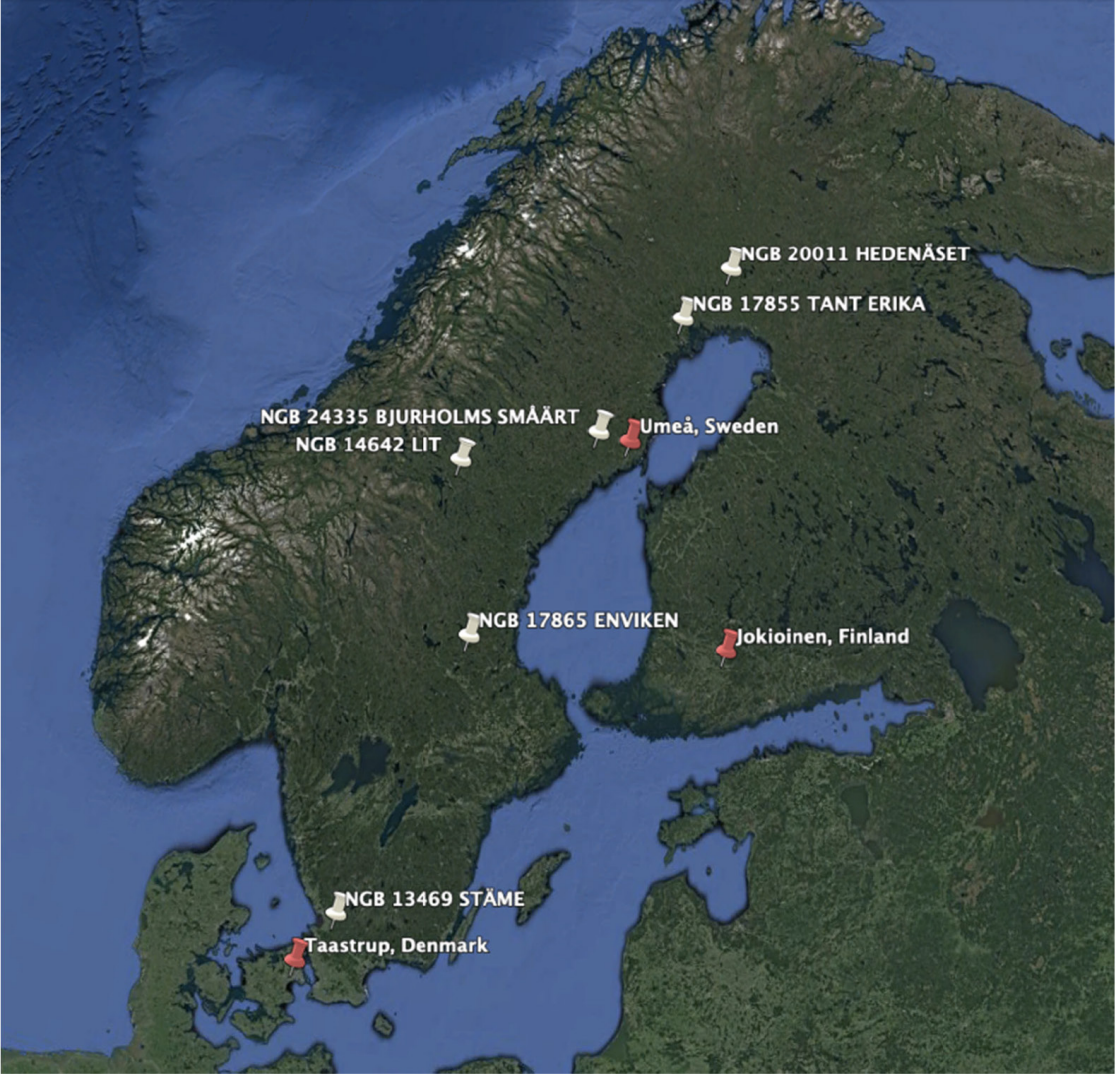
Geographical location of field trial sites (red markers) and the place of origin for the different accessions (white markers).

The cultivation of these pea accessions involved conducting field trials at three distinct locations: Umeå, Sweden; Jokioinen, Finland; and Taastrup, Denmark. These trials were held over a 2‐year period, specifically in 2018 and 2019 (Carlson‐Nilsson et al., 2021). Figure 1 shows both the field trial locations and the origins of the different pea accessions. The selection of these geographical locations and the inclusion of multiple cultivation seasons ensure a comprehensive assessment of the sensory variations and factors that influence the sensory quality of the peas under diverse environmental and climatic conditions.

#### 2.1.2 Harvest conditions and adapted study design

In 2018, peas cultivated in Denmark experienced favorable conditions, resulting in a bountiful yield of fully ripe peas. The relatively high average temperature (18.4°C), significant total radiation (2329.6 MJ/m^2^), and implementation of drip hoses contributed to this success. However, the harvest in Finland in 2018 and in Sweden in 2019 was significantly smaller, and the peas were not fully mature at the time of harvesting. According to the explanation by Carlson‐Nilsson et al. (2021), this outcome can be attributed to low precipitation during the sowing period and early stages of growth, leading to poor and uneven seedling emergence, as seen in Finland in 2018. Similarly, in Sweden in 2019, delayed sowing dates (June 20–24) compared to the previous year affected the ability of the accessions to reach dry seed maturation.

The study design thus had to be modified due to the minimal harvest and the sensory panel's identification of unpleasant qualities like *musty odor* and *metallic taste* in the peas from Finland in 2018 and Sweden in 2019. Consequently, these peas were completely excluded from the design. However, the adapted study design still includes six accessions from three locations, with cultivation spanning two seasons in Denmark and one season each in Finland and Sweden. This modified design ensures a robust assessment of the pea samples across different geographical regions and conditions. Table 2 provides detailed insights, including sowing dates, average temperature, total precipitation, presence of drip hoses, irrigation methods, total radiation, average photosynthetic light period, average photoperiod, and trial duration. Incorporating these data significantly strengthens our analysis and findings.

**TABLE 2:** Climate and irrigation metrics across years and locations (with data from Carlson‐Nilsson et al., 2021).

Locations	Taastrup, Denmark		Jokioinen, Finland		Umeå, Sweden	
Harvest year	2018	2019	2018	2019	2018	2019
Included in the study	Yes	Yes	No	Yes	Yes	No
Sowing date	May 3	May 2	May 24	May 21	May 25–29	June 20–24
Average temperature (°C)	18.4	15.6	17.0	15.6	16.0	14.1
Total precipitation (mm)	62.6	211.3	122.1	223	162.6	175.8
Drip hoses	Yes	Yes	No	No	No	No
Manual irrigation	No	No	When needed	When needed	When needed	When needed
Total radiation (MJ/m^2^)	2329.6	2090.5	2019.9	2108.2	2087.6	1462.4
Average photosynthetic light period (h)	15.7	15.2	15.4	15.0	16.0	14.9
Average photoperiod (h)	16.8	16.7	17.3	17.3	18.3	17.3
Number of days from sowing to end of trial	100	106	112	114	109	89

### 2.2 Method and analysis

#### 2.2.1 Preparing and serving peas

Before proceeding with sensory evaluation, the peas underwent a specific preparation process. They were soaked in water for a duration of 12 h, rinsed thoroughly, and then placed in vacuum‐sealed plastic bags along with fresh water. The ratio used was 1 part peas to 2 parts water, and a 1% salt solution was added relative to the amount of water used. Next, the peas were cooked sous vide in a water bath at a temperature of 90°C for 90 min. Subsequently, they were stored in refrigerators at 6°C (± 2°C) until ready for serving.

In the sensory laboratory (ISO 8589:2007), the peas were presented in white plastic bowls, maintained at a temperature of 20°C (±2°C). The serving sizes ranged from 5 to 10 peas, depending on the size of the pea, and assessors were allowed to determine their own tasting sizes. To cleanse their palates between evaluations, assessors had access to drinking water. It is important to note that this protocol was followed consistently throughout the panel training and evaluation process.

#### 2.2.2 Sensory panel training and generation of attributes

A sensory panel, consisting of 12 undergraduate students from Örebro University's School of Hospitality, Culinary Arts, and Meal Science, willingly volunteered to take part in the sensory panel. Prior to their participation in the study, informed consent was obtained from each participant. The study has been conducted in Sweden. According to the Swedish law governing ethical vetting—the Swedish Act (2003:460) concerning the ethical review of research involving humans—this kind of sensory study does not need ethical approval in Sweden. For the final evaluations, 10 students participated in the first evaluation, and 9 students participated in the second evaluation.

The training program spanned 20 h and comprised 10 sessions conducted over a period of 4 weeks. These sessions encompassed several evaluation techniques, including the check‐all‐that‐apply (CATA) method, ranking tests, and descriptive analysis. The training sessions also involved feedback and discussions to calibrate the sensory panel. The primary goal of this training was to enhance the panel's discrimination abilities and ensure repeatability in their evaluations (Labbe et al., 2004). By employing a diverse range of evaluation methods and fostering active engagement and dialogue, this approach aimed to improve their ability to discern subtle differences between pea samples and ensure consistent and reliable assessments throughout the study.

Table 3 provides an overview of the sensory attributes used, not used, and added during the initial sensory profiling using the CATA method, as well as the three descriptive analysis evaluations conducted throughout the training. During training, the sensory panel identified additional sensory attributes which are not listed in Table 3. These attributes were the following: mint, citrus, silage, green pea, asparagus, green apple, yellow apple, sour odor, pepper, oats, walnut, Brazil nut, acorn, roasted, dairy protein, vegan butter, milk chocolate, chocolate, cacao, musty, meaty, licorice, lard, mushrooms, shiitake, smoky, and pepper.

**TABLE 3:** An overview of the sensory attributes' evolution throughout the sensory panel training, including the attributes used for the final evaluation, indicated by a • symbol.

Sensory attributes	Training				Final evaluation
	CATA	Descriptive I	Descriptive II	Descriptive III	Descriptive IV
Almond	•				
Bitter	•	•	•	•	•
Buttery	•	•	•	•	•
Cacao		•	•	•	•
Cereal	•	•			•
Chestnut	•				
Compact	•				
Compost	•				
Corn	•	•	•	•	•
Crunchy	•				
Dry	•				
Earthy	•	•	•	•	•
Fatty	•				
Fermented		•	•	•	•
Floral			•	•	•
Fruity		•			
Grainy	•	•	•		
Grassy	•				
Hay	•				
Hazelnut	•				
Herby	•	•	•	•	•
Iron	•				
Juicy		•	•		
Metallic	•				
Mineral	•				
Mushroom	•				
Nutty	•	•	•	•	•
Popcorn	•				
Porous	•				
Potato	•				
Roasted			•	•	•
Root fruit	•		•	•	•
Salt	•	•	•	•	•
Soft	•				
Sour	•	•	•	•	•
Spicy	•	•	•	•	•
Stable	•				
Sulfur	•				
Sunflower seed	•				
Sweet	•	•	•	•	•
Tender	•				
Tough	•	•	•		
Umami	•	•	•	•	•
Vegetable	•				
Woody	•				
Yeasty		•			

#### 2.2.3 Sensory evaluation, data analysis, and visualization

For the final evaluation, the sensory panel conducted a quantitative descriptive test to assess the potential impact of accession, location, and year of harvest in two separate sessions. A summary of the sensory attributes utilized in the final sensory profiling can be found in Table 3. In the first session, 10 panelists participated, and the evaluated accessions were “Lit,” “Stäme,” and “Bjurholms småärt.” The second session included nine panelists and focused on “Tant Erika,” “Hedenäset,” and “Enviken.” Each pea accession went through a sensory evaluation, comprising 12 assessments carried out by each panelist. These assessments included peas from three different locations, with one of them including peas from two separate seasons. Moreover, to ensure accuracy and consistency, the evaluations were conducted in two replicates. This approach aimed to provide an understanding of how each pea accession performed under various growing conditions.

The performance of the sensory panel was assessed by analyzing panel discrimination, agreement, and repeatability using the EyeOpenR software (version 4.1.11), Logic8 B.V., Elst, Gelderland, Netherlands; available at https://eyequestion.nl.

To analyze the sensory profiling data, we employed Canonical Variate Analysis by EyeOpenR, a mapping method for visually representing the outcomes of a two‐way multivariate analysis of variance. According to Peltier et al. (2015), this method yields a product map that maximizes product separation while keeping individual evaluations of the same product as close together as possible. We utilized product characterization by EyeOpenR to identify significant differences above and below the mean value for each attribute. For pairwise comparisons, including and excluding the control of the experiment‐wise Type I error rate, we employed ANOVA (Tukey's HSD and Fisher's LSD) analysis.

To further enhance the visual representation, the sensory attributes in the sensory wheels are directly derived from the actual colors of the tested accessions. We collected these color values using an NCS Colorpin II (NCS—Natural Color System©, provided by NCS Color AB, Stockholm, Sweden; available at: https://ncscolour.com/products/ncs‐colourpin‐ii). The utilization of this color‐coded representation adds an extra dimension to the sensory profiles, resulting in a visually appealing and informative display of the distinct attributes of each landrace.

## 3 RESULTS AND DISCUSSION

### 3.1 Sensory panel performance—Discrimination and repeatability

During the final evaluation, which spanned 2 days and took place after the third training session, the performance of the sensory panel was thoroughly evaluated. To ensure data reliability, one panelist was excluded from the analysis due to their unsatisfactory median repeatability in both evaluations. On the other hand, all other panelists (10 during the first day and 9 during the second day) demonstrated *good* median repeatability in both evaluations. The specific results of their individual repeatability are not provided here.

Table 4 provides a breakdown of the sensory attributes used, categorized by odor and taste. The results indicated that the panel was able to distinguish most of the sensory attributes with high significance (*p* < .001), except for *spicy taste* in evaluation II. Furthermore, consistent repeatability was observed for attributes such as *cacao*, *cereal*, *earthy, roasted*, and *root fruit odor*, as well as *cacao*, *corn, herby*, *salt*, *sour*, *sweet*, and *umami taste*, across both evaluations. However, there were poor repeatability in the attributes *nutty taste* and *fermented odor* in evaluations I and II, respectively (as demonstrated in Table 4). The panel acknowledged that the sensory attribute *corn* lacks precision but still contributes a distinct quality beyond being merely *buttery* and *nutty*. Additionally, the evaluation of *salt* taste in peas was noted to be somewhat challenging due to the addition of salt during the boiling process.

**TABLE 4:** Sensory panel discrimination and repeatability.

		Final evaluation I: Lit, Stäme, and Bjurholms småärt		Final evaluation II: Tant Erika, Hedenäset, and Enviken	
Panel summary		Discrimination^a^	Repeatability^b^	Discrimination^a^	Repeatability^b^
Odor	Buttery	<0.001	13.44	<0.001	16.88
	Cacao	<0.001	13.93	<0.001	3.08
	Cereal	<0.001	12.78	<0.001	12.77
	Earthy	<0.001	15.21	<0.001	13.46
	Fermented	<0.001	13.83	<0.001	16.96
	Floral	<0.001	10.5	<0.001	15.05
	Nutty	<0.001	15.84	<0.001	16.17
	Roasted	<0.001	14.39	<0.001	9.24
	Root fruit	<0.001	10.19	<0.001	13.65
Taste	Bitter	<0.001	15.4	<0.001	13.76
	Buttery	<0.001	15.26	<0.001	15.67
	Cacao	<0.001	11.25	0.013	2.89
	Corn	<0.001	9.55	<0.001	14.44
	Herby	<0.001	13.59	<0.001	14.17
	Nutty	<0.001	16.38	<0.001	14.41
	Salt	<0.001	12.7	<0.001	12.96
	Sour	<0.001	11.04	<0.001	9.8
	Spicy	<0.001	15.28	0.325	7.54
	Sweet	<0.001	14.54	<0.001	13.71
	Umami	<0.001	13.91	<0.001	14.53

*Note*: Discrimination is assessed based on *p*‐values, while repeatability is evaluated using mean square error (MSE). To aid understanding, we have color coded the panel's discrimination and repeatability as follows—good is represented by the color green, borderline is indicated by the color yellow, poor is shown in orange, and bad is highlighted in red.

^a^ Panel Discrimination; good (*p* ≤ .05), borderline ( *p* ≤ .10), poor ( *p* ≤ .15), and bad (*p* > .15).

^b^ Panel Repeatability; good (<75th percentile), borderline (75th–90th percentile), poor (90th–95th percentile), and bad (>95th percentile).

### 3.2 Analyzing sensory profiling data by canonical variate analysis

**FIGURE 2:**
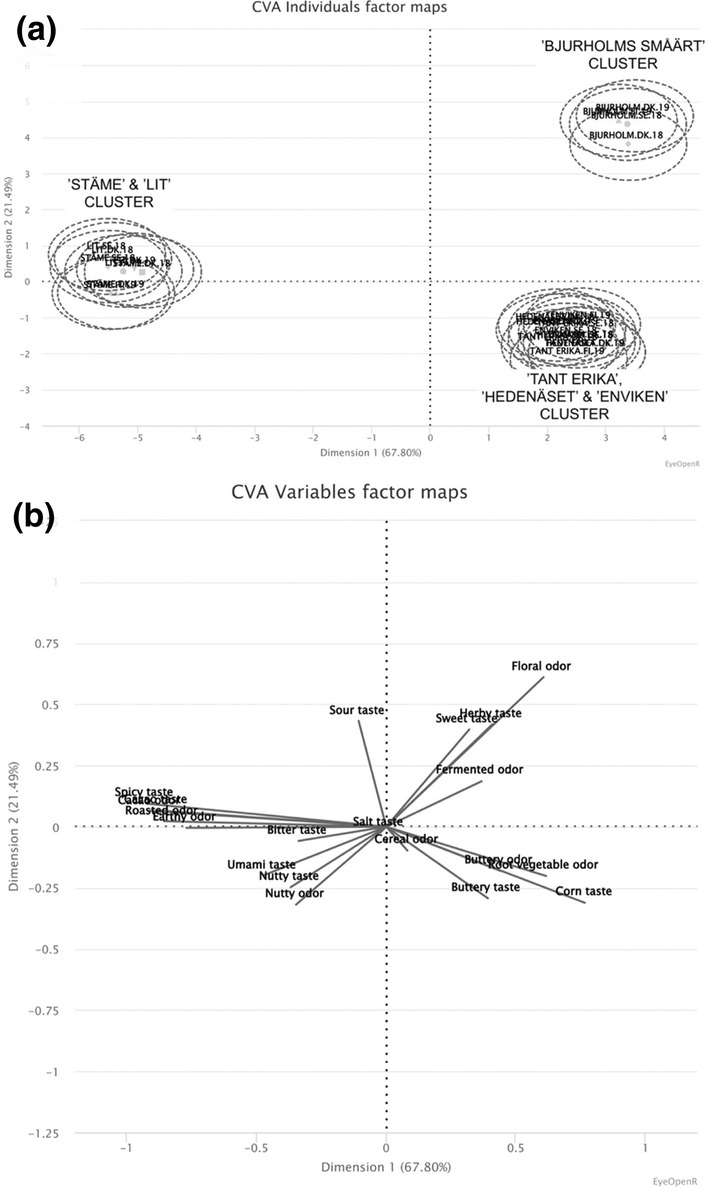
A visual representation of the clustering of pea accessions, based on their sensory attributes, analyzed using canonical variate analysis (CVA). This analysis offer insights into similarities and differences among the pea accessions studied. (a) represents the different pea accessions, positioned within three distinct clusters. The sensory attributes being assessed are visually represented along the axes in (b), providing a view of how these attributes contribute to the overall clustering patterns observed among the pea accessions.

The canonical variate analysis provided valuable insights, revealing three distinct clusters among the pea harvests. The first cluster comprised all harvests of “Bjurholms småärt,” exhibiting higher scores for *floral* and *fermented odor*, as well as *herby* and *sweet taste*. The second cluster consisted of all harvests of “Tant Erika,” “Hedenäset,” and “Enviken,” exhibiting higher scores for *corn* and *buttery taste*, as well as *root fruit, buttery*, and *cereal odor*. Lastly, the third cluster encompassed all harvests of “Lit” and “Stäme,” scoring higher in *spicy, cacao, umami, nutty*, and *bitter taste*, as well as *cacao*, *roasted earthy*, and *nutty odor* (as depicted in Figure 2). These findings suggest that accession holds more significant influence in explaining sensory variations compared to the location or year of harvest.

The clustering of peas can be partially explained by the type of pea cultivar; however, there are exceptions. For example, both “Tant Erika” and “Hedenäset” belong to the sugar pea seed type, but “Enviken” is a shelling pea. In addition to these differences, there are noticeable differences in their sizes. Similarly, “Lit” and “Stäme” are both gray peas (purple color of flower), but they exhibit distinct differences in terms of color, shape, and size. Furthermore, although “Lit,” “Stäme,” and “Bjurholms småärt” fall under the category of field peas, “Bjurholms småärt” stands out due to its unique sensory profile, potentially attributed to its white flowering nature and lower tannin content compared to purple flowering peas. Tannins, known for their astringent taste, contribute to the slightly bitter flavor and reduced sweetness observed in purple flowering peas.

#### 3.2.1 Visualizing sensory profiles by accession and cluster

**FIGURE 3:**
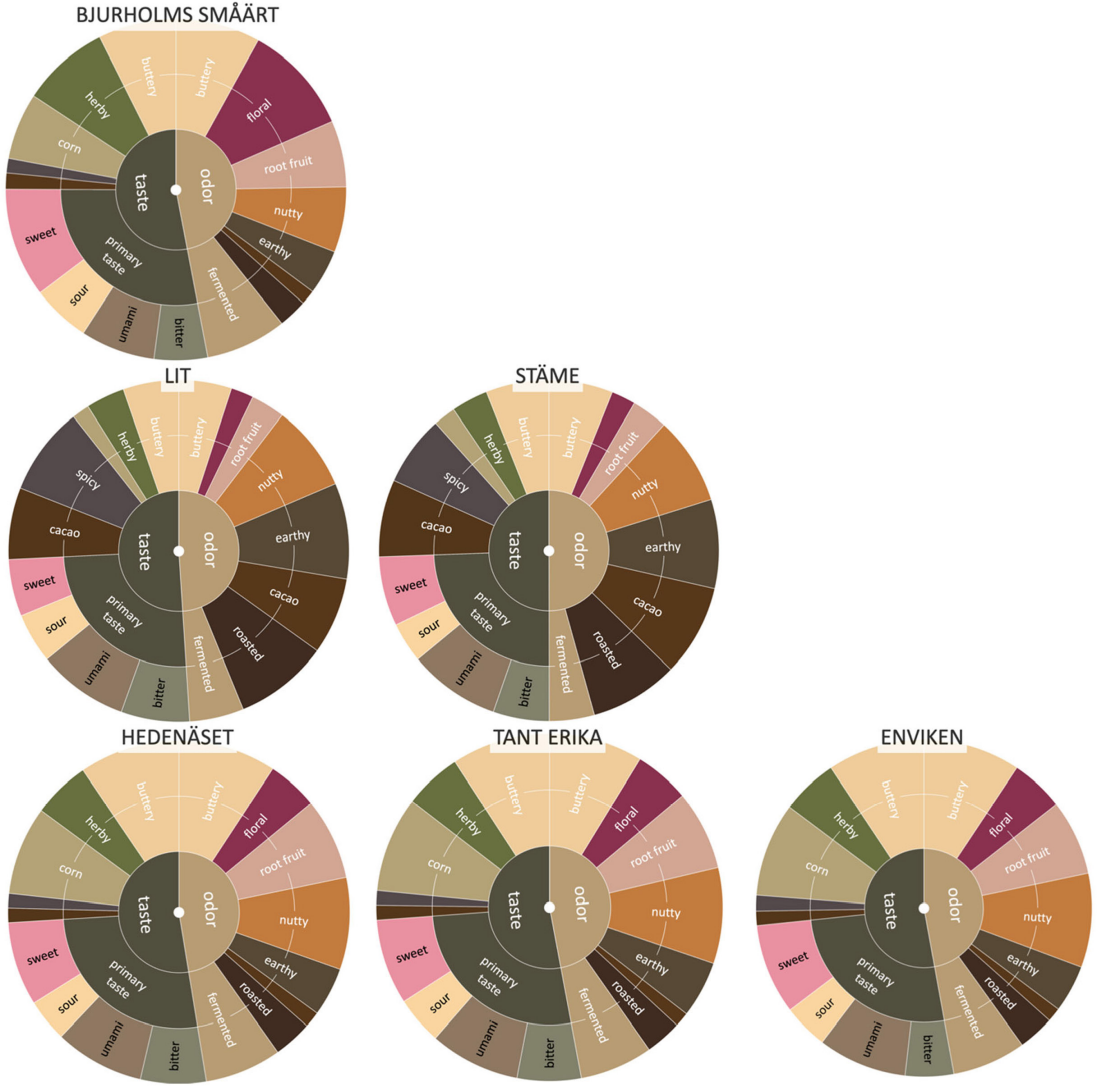
Color‐coded sensory wheels displaying attributes with significant differences (*p* < .05) among the six pea accessions. The wheels are categorized based on odor and taste attributes, with their arrangement representing the three clusters identified through canonical variate analysis. This visual representation facilitates easy identification and comparison of distinct sensory profiles between the pea accessions and clusters.

In Figure 3, the sensory profiles of the six pea accessions are visually represented as sensory wheels, showcasing frequency scores for each attribute. The sensory attributes are grouped according to odor and taste, similar to Table 3. Additionally, the sensory wheels are grouped based on the three distinct clusters generated by the canonical variate analysis (CVA), as shown in Figure 2. This organization facilitates the ease of comparison and interpretation of the sensory attributes within each cluster, providing a understanding of the unique sensory profiles exhibited by the six pea accessions.

The frequency values utilized in the visualization of these wheels were derived from the mean values obtained from 12 assessments conducted by each panelist for every pea accession (as described in Section 2.2.3). To emphasize significant differences, only the sensory attributes that exhibited such differences between the accessions, determined by a *p*‐value of 0.05 using Tukey's HSD, have been included in the sensory wheels. Consequently, the sensory attributes *cereal odor*, *salt taste*, and *nutty taste* were excluded from the analysis as they did not show significant variation.

The sensory profiles of the accessions within clusters displayed only subtle variations, as illustrated in Figure 3. However, through Tukey's HSD analysis, significant differences emerged between certain accessions within clusters. For example, “Stäme” demonstrated a higher level of *sweetness* compared to “Lit,” while “Lit” exhibited a stronger *bitterness* than “Stäme” (*p* value <.01). Moreover, “Enviken” scored higher in terms of *sweetness* compared to “Hedenäset” and “Tant Erika” (*p* value <.05), and it also exhibited a more pronounced *buttery odor* compared to “Tant Erika” (*p* value <.01).

### 3.3 Limited impact of location and harvest year on sensory profiles, with notable exceptions

The canonical variate analysis revealed that the sensory profiles were generally unaffected by location and harvest year, as supported by the ANOVA analysis (Table 5). Despite this trend, a few exceptions were observed, as detailed in Table 6.

**TABLE 5:** Influence of accession, location, and harvest year on all sensory attributes, examined using ANOVA.

Source	df	Sum of squares	Mean squares	*F*	Pr > *F*
Harvest year	1	0,381	0,381	0,001	0,973
Location	2	117,293	58,647	0,177	0,838
Accession	5	24,848,437	4969,687	15	7,1E‐14
Harvest year*location	0	0			
Harvest year*Accession	5	937,835	187,567	0,566	0,726
Location*Accession	10	1281,818	128,182	0,387	0,952

**TABLE 6:** Influence of accession, location, and harvest year on each individual sensory attribute, examined using ANOVA with *p*‐values provided as shown in the table.

Sensory attributes		Accession	Location	Harvest year	Location*Accession	Harvest year*Accession
Odor	Buttery	0.000	0.587	0.794	0.599	0.573
	Cacao	0.000	0.984	0.791	0.961	0.956
	Cereal	0.065	0.382	0.389	0.873	0.868
	Earthy	0.000	0.258	0.522	0.978	0.816
	Fermented	0.000	0.539	0.522	0.985	0.684
	Floral	0.000	0.948	0.886	0.964	0.905
	Nutty	0.000	0.840	0.640	0.952	0.869
	Roasted	0.000	0.972	0.303	0.805	0.754
	Root fruit	0.000	0.786	0.749	0.407	0.852
Taste	Bitter	0.000	0.567	0.452	0.397	0.638
	Buttery	0.000	0.163	0.439	0.339	0.173
	Cacao	0.000	0.591	0.957	0.930	0.999
	Corn	0.000	0.371	0.265	0.908	0.060
	Herby	0.000	0.926	0.672	0.962	0.808
	Nutty	0.000	0.571	0.905	0.993	0.895
	Salt	0.906	0.260	0.546	0.826	0.700
	Sour	0.000	0.055	0.890	0.888	0.207
	Spicy	0.000	0.822	0.283	0.934	0.939
	Sweet	0.000	0.820	0.428	0.725	0.212
	Umami	0.000	0.087	0.317	0.802	0.133

One such exception was the *sour taste* attribute, for which there is an indication of a potential influence of location on the sour taste (*p* = .055). Likewise, there also appears to be some influence of location on the *umami taste*, although this effect is less pronounced than for the *sour taste* (*p* = .087). These observations hint that certain sensory attributes like *sour taste* and *umami taste* may be more sensitive to environmental variations compared to others. This could guide further research to explore these potential effects in more detail.

Additionally, there might be some combined effect of accession and harvest year on the perceived *corn taste* (*p*‐value .06). Such an interaction could imply that different accessions may respond to variations in harvest year differently in terms of their *corn taste* profile, and it might be worth further investigation to ascertain whether there are meaningful trends or patterns contributing to this near‐significant result.

Overall, while the impact of location and harvest year on sensory profiles was limited, these exceptions provide insights into the potential influences on specific sensory attributes.

#### 3.3.1 Possible influence of light conditions, temperature, and soil on landrace peas

Even though light conditions related to latitude and temperature have been shown to influence the sensory quality of broccoli florets, such as lower *bitterness* in the northernmost location (Johansen et al., 2017), our study found that *bitterness* was high in peas cultivated in the northernmost locations of Finland and Sweden. For example, “Lit” and “Enviken” cultivated in Finland in 2019, and “Bjurholm” cultivated in Sweden in 2018 exhibited high *bitterness* levels. These findings align with the conclusions of Roland et al. (2017) regarding variations in saponin content among pea accessions and studies.

Mølmann et al. (2018) suggest that high‐latitude light conditions with long photoperiods can contribute to *sweeter* and less *bitter taste* in swede roots (*Brassica napus*). In our study, Denmark 2019 had a lower average photoperiod (16.7 h) compared to Finland 2019 (17.3 h) (Carlson‐Nilsson et al., 2021). The different phenological phases also occurred at different time points, meaning that, for example, the difference in photoperiod was even larger during flowering and early pod development. Interestingly, “Stäme” cultivated in Denmark in 2019 showed a significantly higher *sour taste* compared to “Stäme” cultivated in Finland in 2019 (*p* value < .05) according to Fischer's LSD. Moreover, “Enviken” cultivated in Denmark in 2019 exhibited a significantly higher *sour taste* compared to “Enviken” cultivated in Denmark in 2018 (*p* value <.05), despite having similar average photoperiods. It is worth noting that the average temperature in Denmark in 2019 was higher (18.4°C on average) compared to Finland in 2019 and Denmark in 2018 (15.6°C for both) (Carlson‐Nilsson et al., 2021). This difference in temperature might be a possible explanation for the lower *sour taste* in “Enviken” cultivated in Denmark in 2018, which should be further investigated by northern gardeners and farmers. Even though the *sour taste* did not have a significant Location*Accession or Harvest Year*Accession interaction in the ANOVA (Table 6), these significant differences in *sour taste* between specific conditions (Denmark 2019 vs. Finland 2019 and Denmark 2019 vs. Denmark 2018) for certain accessions (“Stäme” and “Enviken”) can exist even if interaction effects are not significant overall.

*Umami taste* has been suggested as an important component in plant‐based raw ingredients (Mouritsen & Styrbæk, 2020). In our study, “Lit” and “Stäme,” field peas with colored flowers, scored the highest intensity of *umami taste*. Specifically, “Stäme” cultivated in Denmark in 2019 scored the highest, significantly higher (*p* value <.01) than “Lit” cultivated in Denmark in 2019, “Lit” cultivated in Finland in 2019, and “Stäme” cultivated in Sweden in 2018 according to Fischer's LSD. Moreover, “Lit” cultivated in Denmark in 2018 scored the next highest, significantly higher (*p* value <.05) than “Lit” cultivated in Denmark in 2019 and “Lit” cultivated in Finland in 2019, according to Fischer's LSD. On the other hand, “Bjurholms småärt,” “Tant Erika,” “Hedenäset,” and “Stäme” cultivated in Sweden in 2018 scored the lowest on *umami taste* compared to the same accessions cultivated in Denmark in 2018, Denmark in 2019, and Finland in 2019. Unfortunately, no explanation for this discrepancy based on weather and climate factors could be found in the available data from the field trials. One potential hypothesis to explore in future studies is the influence of soil amino acid concentration on the perceived taste of *umami* in peas (Sauheitl et al., 2009).

## 4 CONCLUSION

The sensory variation in the studied landrace pea accessions is primarily attributed to the chosen accession, despite the differences in location and harvest year. Factors such as temperature, photoperiod, precipitation, and soil vary, yet the sensory profiling reveals that the harvests of the same variety are remarkably similar. While the location and harvest year have relatively small effects on the overall sensory profile, there is a potential influence observed in the specific *sour taste* and, to a lesser extent, *umami taste*, which indicates the impact of the location. These findings contribute to our understanding of how genetic diversity, location, and harvest year collectively shape the sensory profile of peas. They emphasize the importance of careful variety selection and breeding in enhancing the sensory experience of peas while recognizing the potential for distinct flavors associated with different locations. This knowledge can guide future breeding and cultivation strategies, ultimately leading to peas with enhanced sensory qualities and wider appreciation in various culinary applications. Future research should investigate specific terroir effects, such as temperature and soil composition, which were not controlled in our study, to understand their influence on the sensory profiles of landrace peas.

## AUTHOR CONTRIBUTIONS

**Magnus Westling:** Conceptualization (equal); data curation (equal); formal analysis (lead); investigation (equal); methodology (equal); visualization (lead); writing – original draft (lead); writing – review and editing (equal). **Matti Wiking Leino:** Conceptualization (equal); funding acquisition (equal); investigation (equal); methodology (equal); project administration (equal); resources (equal); writing – review and editing (equal). **Stefan Wennström:** Formal analysis (supporting); investigation (supporting); supervision (equal); validation (equal); writing – review and editing (equal). **Åsa Öström:** Conceptualization (equal); data curation (equal); formal analysis (equal); funding acquisition (lead); investigation (equal); methodology (equal); project administration (lead); resources (lead); software (lead); supervision (lead); validation (equal); writing – review and editing (equal).

## CONFLICT OF INTEREST STATEMENT

None declared.

## Data Availability

The data that support the findings of this study are available from the corresponding author upon reasonable request.
